# Quantum-Inspired Magnetic Hamiltonian Monte Carlo

**DOI:** 10.1371/journal.pone.0258277

**Published:** 2021-10-05

**Authors:** Wilson Tsakane Mongwe, Rendani Mbuvha, Tshilidzi Marwala

**Affiliations:** 1 School of Electrical Engineering, University of Johannesburg, Johannesburg, South Africa; 2 School of Statistics and Actuarial Science, University of Witwatersrand, Johannesburg, South Africa; Central State University & Ohio University, UNITED STATES

## Abstract

Hamiltonian Monte Carlo (HMC) is a Markov Chain Monte Carlo algorithm that is able to generate distant proposals via the use of Hamiltonian dynamics, which are able to incorporate first-order gradient information about the target posterior. This has driven its rise in popularity in the machine learning community in recent times. It has been shown that making use of the energy-time uncertainty relation from quantum mechanics, one can devise an extension to HMC by allowing the mass matrix to be random with a probability distribution instead of a fixed mass. Furthermore, Magnetic Hamiltonian Monte Carlo (MHMC) has been recently proposed as an extension to HMC and adds a magnetic field to HMC which results in non-canonical dynamics associated with the movement of a particle under a magnetic field. In this work, we utilise the non-canonical dynamics of MHMC while allowing the mass matrix to be random to create the Quantum-Inspired Magnetic Hamiltonian Monte Carlo (QIMHMC) algorithm, which is shown to converge to the correct steady state distribution. Empirical results on a broad class of target posterior distributions show that the proposed method produces better sampling performance than HMC, MHMC and HMC with a random mass matrix.

## 1 Introduction

The inference of complex probabilistic models using Markov Chain Monte Carlo (MCMC) algorithms has become very common [1–7]. MCMC methods have been successfully applied in a variety of fields including health, finance and cosmology [1, 4, 5, 8–15]. The goal of MCMC methods is to construct a Markov chain that leaves the target posterior distribution invariant [3, 16]. This allows the generated samples from the chains to be used to estimate expectations with respect to the target posterior distribution. Examples of such constructions include the Metropolis-Hasting [17] method, slice sampling [18] and Gibbs sampling [19]. While these algorithms are able to construct Markov chains that asymptotically converge to the correct distribution, they still suffer from random-walk behaviour [3]. This results in high correlations between the generated samples and consequently low effective sample sizes.

For instances where the target posterior is differentiable on the Euclidean manifold, Hamiltonian Monte Carlo (HMC) provides a powerful mechanism to sample from differentiable target posterior distributions [20, 21]. HMC extends the parameter space into the phase space via the introduction of an auxiliary momentum variable which ensures that different energy levels are explored. HMC exploits the first-order gradient information of the target density to guide its exploration of the phase space. The use of gradient information reduces the random-walk behaviour typically associated with the Metropolis-Hastings algorithm [3, 10, 21]. HMC introduces the mass matrix for the momentum variable, trajectory length and step size parameters that need to be tuned for optimal results. Extensions of HMC include Riemannian Manifold Hamiltonian Monte Carlo (RMHMC) [1], the No-U-Turn Sampler (NUTS) [22] and Magnetic Hamiltonian Monte Carlo (MHMC) [6]. Furthermore, machine learning techniques have also been utilised to enhance the efficiency of Monte Carlo sampling algorithms. In Mcnaughton *et al*. [23], autoregressive neural networks are employed to boost the efficiency of Monte Carlo methods, while Levy *et al*. [24] generalises Hamiltonian Monte Carlo using neural networks.

RMHMC extends HMC by incorporating second-order gradient information of the target posterior. This allows RMHMC to take into account the local geometry of the target as it explores the phase space, which is important for ill-conditioned distributions [1, 5, 7, 25]. This results in the Hamiltonian being non-separable, which necessitates the use of implicit numerical integration schemes which are computationally expensive. On the other-hand, NUTS extends HMC by automatically tuning the trajectory length and step size parameters of HMC, which would need to be specified by the user [22, 25]. The trajectory length is tuned by iteratively doubling the trajectory length until either the parameters trace back, or the Hamiltonian becomes infinite [22]. The step size is tuned by employing the dual averaging methodology during the burn-in phase [22]. This removes the need for manual tuning of these parameters and the associated costly pilot runs, making HMC more accessible to non-experts. A recent extension to HMC is MHMC which adds a magnetic field to HMC [6]. This results in faster convergence to the correct target density, and much lower auto-correlations between the generated samples when compared to HMC [4, 6, 26]. When the magnetic component of MHMC is absent, MHMC and HMC have the same dynamics [4, 6, 26]. MHMC has similar execution times to HMC, which illustrates the close relationship between HMC and MHMC.

One of the parameters that needs to be set in HMC is the mass matrix of the auxiliary momentum variable. This mass matrix is typically set to equal the identity matrix [1, 6, 9, 21]. Although this produces good results, it is not necessarily the optimal choice across all target distributions. In RMHMC, the mass matrix is set to be the Hessian of the negative log-density—which is the fisher information metric [1, 25]. In Quantum-Inspired Hamiltonian Monte Carlo algorithm (QIHMC) [7], the mass matrix is set to be a stochastic process. This is motivated by the energy-time uncertainty relation from quantum mechanics, which allows a particle’s mass to be stochastic to rather fixed [7].

QIHMC has been shown to improve performance when sampling from a broad class of distributions which occur in sparse modeling via bridge regression, image denoising and Bayesian neural network pruning [5, 7]. This is particularly important for spiky and multi-modal distributions, where HMC is inefficient [5, 7]. The use of a random mass matrix is yet to be considered in the context of MHMC in the literature. Given that MHMC is closely related to HMC, one would expect that making the mass matrix random would result in improved sampling performance when compared to MHMC with fixed mass.

In this work, we present the Quantum-Inspired Magnetic Hamiltonian Monte Carlo (QIMHMC) algorithm which uses a random mass matrix in the non-canonical Hamiltonian dynamics offered by MHMC. A proof that the proposed algorithm converges to the correct distribution is presented. Numerical experiments across various benchmarks including the Banana shaped distribution, multivariate Gaussian distributions and Bayesian logistic regression show that the proposed algorithm outperforms HMC, MHMC and QIHMC. The main contributions of this work can be summarised as:

- We present the Quantum-Inspired Magnetic Hamiltonian Monte Carlo which employs a stochastic mass matrix in Magnetic Hamiltonian Monte Carlo.
- We prove that the proposed algorithm converges to the correct target density.
- Numerical experiments on various target posteriors show that the proposed method outperforms Hamiltonian Monte Carlo, Magnetic Hamiltonian Monte Carlo and Quantum-Inspired Hamiltonian Monte Carlo.

The remainder of this paper is structured as follows: Section 2 discusses the Markov Chain Monte Carlo methods that form the basis of the new method, Section 3 presents the proposed method, Section 4 outlines the target distributions considered, Section 5 outlines the experiments conducted, Section 6 presents and discusses the results of the experiments and we provide a conclusion in Section 7.

## 2 Hamiltonian Monte Carlo methods

Instead of directly sampling the parameters, Hamiltonian Monte Carlo (HMC) introduces an auxiliary momentum variable and then samples the parameters and momenta jointly. HMC improves upon the Metropolis-Hastings [17] algorithm by using first-order gradient information of the target to guide its exploration [9, 20], which leads to low auto-correlations between the generated samples. The parameter vector **w** and momentum variable **p**, which is typically chosen to be independent of **w**, form a dynamic system whose Hamiltonian is written as:

$$\begin{array}{c} \text{H}(w,p)=\text{U}(w)+\text{K}(p) \end{array}$$ (1)

where U(**w**) is the negative log-likelihood of the target posterior distribution and K(**p**) is the kinetic energy defined by the kernel of a Gaussian with mass matrix **M** [10]:

$$\begin{array}{c} \text{K}\left(p\right)=\frac{1}{2}\text{log}\;({\left(2\pi \right)}^{D}\left|M\right|)+\frac{p^{\text{T}}M^{-1}p}{2}. \end{array}$$ (2)

The equations governing the Hamiltonian dynamics step are defined by Hamilton’s equations at a fictitious time *t* as follows [8, 20]:

$$\begin{array}{c} \frac{\text{d}w}{\partial t}=\frac{\partial \text{H}(w,p)}{\partial p};\;\frac{\text{d}p}{\partial t}=-\frac{\partial \text{H}(w,p)}{\partial w}. \end{array}$$ (3)

The evolution of this Hamiltonian system must preserve both volume and total energy [3, 21]. As the Hamiltonian in Eq (1) is separable, to traverse the space we can employ the leapfrog integrator [8, 20]. The update equations for the leapfrog integration scheme are:

$$\begin{array}{c} \begin{array}{cc} p_{t+\frac{\varepsilon }{2}} & =p_{t}+\frac{\varepsilon }{2}\frac{\partial H(w_{t},p_{t})}{\partial w}\\ w_{t+\varepsilon } & =w_{t}+\varepsilon M^{-1}p_{t+\frac{\varepsilon }{2}}\\ p_{t+\varepsilon } & =p_{t+\frac{\varepsilon }{2}}+\frac{\varepsilon }{2}\frac{\partial H(w_{t+\varepsilon },p_{t+\frac{\varepsilon }{2}})}{\partial w}. \end{array} \end{array}$$ (4)

Due to the discretisation errors arising from the leapfrog integration, HMC utilises the Metropolis-Hastings algorithm in which the parameters w* are accepted with probability:

$$\begin{array}{c} \text{P}\left(\text{accept}\;w^{*}\right)=\text{min}\;(1,\frac{\text{exp}\;(-\text{H}(w^{*},p^{*}))}{\text{exp}\;(-\text{H}(w,p))}). \end{array}$$ (5)

The overall HMC sampling process follows a Gibbs sampling scheme, where we sample the momentum and then sample a new set of parameters given the drawn momentum. Algorithm 1 shows the pseudo-code for the HMC where *ε* is the discretisation step size and *L* is the trajectory length.

**Algorithm 1**: Hamiltonian Monte Carlo

  **Input**: *N*, *ε*, *L*, *w*_init_, *H*(*w*, *p*)

  **Output**: ${\left(w\right)}_{m=0}^{N}$

1: *w*_0_ ← *w*_init_

2: **for** *m* → 1 **to** *N* **do**

3:  $p_{m-1}∼N\left(0,M\right)$

4:  *p*_*m*_, *w*_*m*_ = **Leapfrog**(*p*_*m*−1_, *w*_*m*−1_, *ε*, *L*, *H*) in Eq (4)

5:  *δH* = *H*(*w*_*m*−1_, *p*_*m*−1_) − *H*(*w*_*m*_, *p*_*m*_)

6:  *α*_*m*_ = min(1, exp(*δH*))

7:  *u*_*m*_ ∼ Unif(0, 1)

8:  *w*_*m*_ = **Metropolis**(*α*_*m*_, *u*_*m*_, *w*_*m*_, *w*_*m*−1_) in Eq (5)

9: **end for**

Although HMC improves on the Metropolis-Hastings algorithm and reduces random-walk behaviour, it still produces relatively high auto-correlations in the samples. Magnetic Hamiltonian Monte Carlo (MHMC) is a special case of non-canonical HMC corresponding to motion of a particle in a magnetic field [6, 27], which aims to improve the exploration of the target distribution [6]. This magnetic field adds an extra degree of freedom and can be tuned to attain significantly better sampling performance compared to HMC with very little extra computational cost [4, 6, 26].

MHMC utilises the same Hamiltonian as in HMC, but exploits non-canonical Hamiltonian dynamics where the canonical matrix now has a non-zero element on the diagonal [4, 6]. The MHMC dynamics are:

$$\begin{array}{c} \frac{d}{dt}[\begin{array}{c} \text{w}\\ \text{p} \end{array}]=[\begin{array}{cc} 0 & I\\ -I & G \end{array}][\begin{array}{c} \nabla_{w}\text{H}\left(\text{w},\text{p}\right)\\ \nabla_{p}\text{H}\left(\text{w},\text{p}\right) \end{array}] \end{array}$$ (6)

where **G** is the magnetic field. The update equations for the leapfrog-like integration scheme in MHMC are given as [6]:

$$\begin{array}{c} \begin{array}{cc} p_{t+\frac{\varepsilon }{2}} & =p_{t}+\frac{\varepsilon }{2}\frac{\partial H(w_{t},p_{t})}{\partial w}\\ w_{t+\varepsilon } & =w_{t}+G^{-1}(\text{exp}\;(G\varepsilon )-I)M^{-1}p_{t+\frac{\varepsilon }{2}}\\ p_{t+\frac{\varepsilon }{2}} & =\text{exp}\;(G\varepsilon )p_{t+\frac{\varepsilon }{2}}\\ p_{t+\varepsilon } & =p_{t+\frac{\varepsilon }{2}}+\frac{\varepsilon }{2}\frac{\partial H(w_{t+\varepsilon },p_{t+\frac{\varepsilon }{2}})}{\partial w}. \end{array} \end{array}$$ (7)

Eq (7) shows that MHMC only differs from HMC dynamics through the presence of a non-zero **G** [4, 6]. MHMC and HMC have exactly the same dynamics when the magnetic component is absent. This can be seen by performing a matrix Taylor series expansion of exp(**G** *ε*) in the **w**_*t*+*ε*_ update in Eq (7) and then setting **G** = 0.

As with HMC, these non-canonical dynamics in MHMC cannot be integrated exactly, and a numerical integration scheme with a Metropolis-Hastings acceptance step must be utilised to ensure detailed balance. The algorithm for MHMC is the same as the HMC algorithm in Algorithm 1, except that the leapfrog integration scheme is replaced by the leapfrog-like integration scheme corresponding to MHMC in Eq (7).

An issue that we are yet to address is the selection of the mass matrix **M** in both HMC and MHMC. In practice, **M** is commonly set to equal the identity matrix **I** [4, 6, 10]. Although this produces good results, this approach is not always the optimal approach to use [7]. In this work, we set **M** to be a stochastic process. When applied to HMC, this approach gives result to the Quantum-Inspired Hamiltonian Monte Carlo of Liu and Zhang [7]. A contribution of this work is that we utilise a random mass matrix in MHMC to create the Quantum-Inspired Hamiltonian Monte Carlo (QIMHMC) algorithm which we describe in the following section.

## 3 Quantum-Inspired Magnetic Hamiltonian Monte Carlo

We now present the Quantum-Inspired Magnetic Hamiltonian Monte Carlo (QIMHMC) method which employs a random mass matrix in MHMC. This proposed algorithm has the same dynamics as MHMC in Eqs (6) and (7), with the exception that the mass matrix is random and is re-sampled before generating the auxiliary momentum variable. The algorithmic description of QIMHMC is presented in Algorithm 2.

The proposed algorithm has been named Quantum-Inspired Magnetic Hamiltonian Monte Carlo as it utilises a random mass matrix, which is consistent with the behaviour of quantum particles. In quantum mechanics, a particle can have a mass which is random and has its own distribution, while in classical mechanics a particle has a fixed mass. The classical version of the proposed algorithm is the Magnetic Hamiltonian Monte Carlo method. When a random mass is utilised as inspired by quantum particles, the result is Quantum-Inspired Magnetic Hamiltonian Monte Carlo. Furthermore, this naming convention in consistent with that used by Liu and Zhang [7] for Hamiltonian Monte Carlo. Their work differs with ours in that now the quantum particle is subjected to a magnetic field as well.

As we have introduced another source of randomness in the form of a random mass matrix into MHMC, we need to show that the proposed algorithm converges to the correct steady state distribution. This is provided in Theorem 3.1, which guarantees that the proposed algorithm produces the correct steady state distribution [7].

**Theorem 3.1**. *Consider continuous-time Hamiltonian dynamics with a deterministic time-varying positive-definite mass matrix***M**(*t*) *in*Eq (7). *The marginal density π*_**w**_(**w**) ∝ exp(−*U*(**w**)) *is a unique steady state distribution in the* **w** *space if momentum re-sampling steps* $p_{p}\left(p\right)\propto \text{exp}\;(\frac{p^{\text{T}}M{\left(t\right)}^{-1}p}{2})$ *are included*.

*Proof*. Consider the joint distribution of (**w**, **p**, **M**) given by *π*(**w**, **p**, **M**). Here we have dropped the explicit dependence of **M** on *t* because **M**(*t*) obeys the mass distribution **P**_**M**_(**M**) for all *t*. Employing Bayes theorem we have that:

$$\begin{array}{c} \begin{array}{cc} \pi (w,p,M) & =p(w,p|M)P_{M}\left(M\right)\\ \pi (w,p,M) & \propto \text{exp}\left(-U\left(w\right)\right)\;\text{exp}\;(\frac{p^{\text{T}}M^{-1}p}{2})\\ \Rightarrow \pi (w) & =\int_{p}\int_{M}\pi (w,p,M)dwdM\\ \pi (w) & \propto \text{exp}(-U(w)) \end{array} \end{array}$$ (8)

which means that the marginal steady state distribution *π*(**w**) is the correct posterior distribution.

An aspect that we are yet to address is which distribution **P**_**M**_(**M**) should be used for the mass matrix. This is still an open area of research [5, 7]. In this paper, we consider the simple case where **M** is a diagonal matrix with the entries being sampled from a log-normal distribution where the mean is zero and the standard deviation, which we refer to as the vol-of-vol, is equal to a tunable parameter *α*. We present the sensitivity analysis to the chosen value of *α* in Section 5.3.

Note that the algorithm for QIHMC used in this paper is the same as that outlined in Algorithm 2, except that in step 5 we use the leapfrog integration scheme in Eq (4).

**Algorithm 2**: Quantum-Inspired Magnetic Hamiltonian Monte Carlo

  **Input**: *N*, *ε*, *L*, *w*_init_, *H*(*w*, *p*), **G**

  **Output**: ${\left(w\right)}_{m=0}^{N}$

1: *w*_0_ ← *w*_init_

2: **for** *m* → 1 **to** *N* **do**

3:  **M** ∼ **P**_**M**_(**M**) ← **re-sample mass matrix**

4:  $p_{m-1}∼N\left(0,M\right)$

5:  *p*_*m*_, *w*_*m*_ = **Integrator**(*p*_*m*−1_, *w*_*m*−1_, *ε*, *L*, *H*, **G**) in Eq (7)

6:  *δH* = *H*(*w*_*m*−1_, *p*_*m*−1_) − *H*(*w*_*m*_, *p*_*m*_)

7:  *α*_*m*_ = min(1, exp(*δH*))

8:  *u*_*m*_ ∼ Unif(0, 1)

9:  *w*_*m*_ = **Metropolis**(*α*_*m*_, *u*_*m*_, *w*_*m*_, *w*_*m*−1_) in Eq (5)

10: **end for**

## 4 The target posteriors

In this section, we outline the target distributions that we considered in this work. These target posterior densities have been extensively used in the literature [1, 4–6, 12, 28, 29], including the seminal work of Girolami and Caldehead [1].

### 4.1 Banana shaped distribution

The Banana shaped density is a 2-dimensional non-linear target which was first presented in Haario *et al*. [30]. The likelihood and prior distributions are given as:

$$\begin{array}{c} y|w∼N\left(w_{1}+w_{2}^{2}=1,\sigma_{y}^{2}\right),\;w_{1},w_{2}∼N\left(0,\sigma_{w}^{2}\right) \end{array}$$ (9)

We generated one hundred data points for *y* with $\sigma_{y}^{2}=4$ and $\sigma_{w}^{2}=1$. Due to independence of the data and parameters, the posterior distribution is proportional to:

$$\begin{array}{c} \prod_{i=1}^{i=N}p\left(y_{k}|w\right)p\left(w_{1}\right)p\left(w_{2}\right). \end{array}$$ (10)

where *N* = 100 is the number of observations.

### 4.2 Multivariate Gaussian distributions

The objective is to sample from *D*-dimensional Gaussian distributions N(0,Σ). The covariance matrix Σ is set to be diagonal, with the standard deviations simulated from a log-normal distribution with mean zero and unit standard deviation. In this work, we consider the number of dimensions *D* to be in the set {50, 100}.

### 4.3 Bayesian logistic regression

We model the real world binary classification datasets shown in Table 1 using Bayesian logistic regression. The negative log-likelihood *l*(D|*w*) function associated with logistic regression is given by:

$$\begin{array}{c} l\left(\text{D}|w\right)=\sum_{i}^{N}y_{i}\text{log}\left(w^{T}x_{i}\right)+\left(1-y_{i}\right)\text{log}\left(1-w^{T}x_{i}\right) \end{array}$$ (11)

where D is the data and *N* is the number of observations. Thus, the target unnormalised posterior log distribution is given as:

$$\begin{array}{c} \text{ln}\;p(w|\text{D})=l(\text{D}|w)+\text{ln}\;p(w|\beta ) \end{array}$$ (12)

where ln *p*(**w**|*β*) is the log of the prior distribution on the parameters given the hyperparameters *β*. We model the parameters **w** as having a Gaussian prior with each parameter having zero mean and standard deviation *β* = 10. These settings are the same as those used in Girolami and Caldehead [1].

**Table 1:** Real world benchmark datasets. *N* represents the number of observations. *D* represents the number of model parameters.

Dataset	Features	*N*	*D*
Heart	13	270	14
Australian credit	14	690	15
German credit	24	1 000	25

## 5 Experimental setup

We now outline the settings used for the experiments, the performance metrics used and we perform a sensitivity analysis to the vol-of-vol parameter *α*.

### 5.1 Settings

In all our experiments, we compare the performance of QIMHMC to HMC, MHMC and QIHMC using the following metrics: acceptance rate of the generated samples, the effective sample size and the effective sample size normalised by execution time.

The acceptance rate metric measures the number of generated samples that are accepted in the Metropolis-Hastings acceptance step of the algorithm. The higher the number of accepted samples for the same step size, the more preferable the method. The effective sample size metric is a commonly used metric for assessing the sampling efficiency of an MCMC algorithm. It indicates the number of effectively uncorrelated samples out of the total number of generated samples. The larger the effective sample size, the better the performance of the MCMC method. The effective sample size normalised by execution time metric takes into account the computational resources required to generate the samples and penalises MCMC methods that require more computational resources to generate the same number of uncorrelated samples. The larger this metric, the better the efficiency of algorithm.

The vol-of-vol parameter *α* is set depending on the particular target density. The sensitivity analysis for *α* is presented in Section 5.3. We set *α* to 0.1 for the Banana shaped distribution and *α* was set to 0.3 for the other targets.

The trajectory length for all the MCMC methods considered in this work was set to 100 across all the targets. For the Banana shaped distribution, we used a step size of 0.1 for all the MCMC methods. Step sizes of 0.07 and 0.05 were used for each value of *D*, in that order, for the multivariate Gaussian distributions, with the Bayesian logistic regression datasets all using a step size of 0.02. Ten independent chains were run for each method on each target distribution. A total of 3 000 samples were generated for each target, with the first 1 000 samples discarded as burn-in. This was sufficient for all the algorithms to converge on all the targets. All experiments were conducted on a 64bit CPU using PyTorch.

As mentioned previously, the matrix **G** in the MHMC and QIMHMC methods provides an extra degree of freedom which results in better sampling behavior than HMC [4, 6, 31]. It is not immediately clear how this matrix should be set—this is still an open area of research [4, 6, 27]. In this work, we select only a few dimensions to be influenced by the magnetic field. In particular, **G** was set such that **G**_**1i**_ = *g*, **G**_**i1**_ = −*g* and zero elsewhere for *g* = 0.2. These settings are used across all the targets.

It is worth highlighting that, in this work, the mass matrix **M** is set to the identity matrix for the HMC and MHMC methods. This is what is commonly used in practice [3, 4, 6, 26].

### 5.2 Multivariate effective sample size

This work employs the multivariate effective sample size metric developed by Vats *et al*. [32] instead of the minimum univariate ESS metric typically used in analysing MCMC results. The minimum univariate ESS measure is not able to capture the correlations between the different parameter dimensions, while the multivariate ESS metric is able to incorporate this information [1, 4, 5, 32]. The minimum univariate ESS calculation results in the estimate of the ESS being dominated by the parameter dimensions that mix the slowest, and ignoring all other dimensions [4, 5, 32]. The multivariate ESS is calculated as:

$$\begin{array}{c} \text{ESS}=n\times {\left(\frac{\left|\Lambda \right|}{\left|\Sigma \right|}\right)}^{\frac{1}{D}} \end{array}$$ (13)

where *n* is the number of generated samples, *D* is the number of parameters, Λ is the sample covariance matrix and Σ is the estimate of the Markov chain standard error. When *D* = 1, the multivariate ESS is equivalent to the univariate ESS measure.

It is worth noting that for the case where *D* > 1, the disadvantage of the multivariate ESS approach is with regards to the estimation of Λ and Σ. If non-stable approaches are used, they can lead to unreliable results [32]. This is particularly important when *D* is large.

### 5.3 Sensitivity to the vol-of-vol

**Fig 1:**
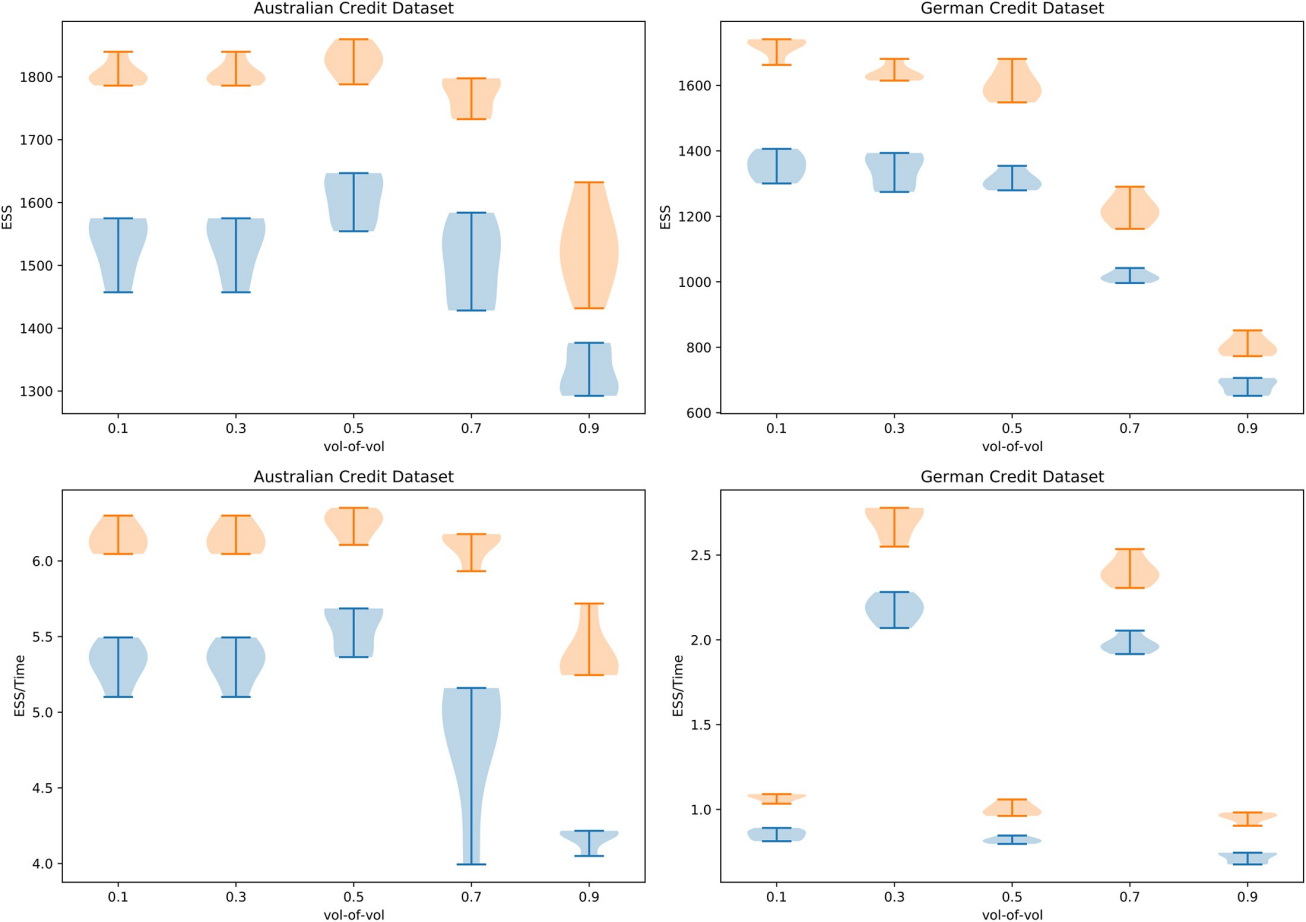
ESS and ESS/*t* for ten runs of QIHMC (blue) and QIMHMC (orange) on the Australian and German credit datasets with varying choices of the vol-of-vol parameter.

In this section, we present the sensitivity analysis for the chosen vol-of-vol parameter *α*. We considered values of *α* ∈ {0.1, 0.3, 0.5, 0.7, 0.9}, with all the other settings being the same as those outlined in Section 5.1. The results are presented in Fig 1. The results show that the ESS has a tendency of decreasing for both QIHMC and QIMHMC with increasing *α* on both the Australian and German credit datasets. Furthermore, QIMHMC outperforms QIHMC for all values of *α* on both an ESS and normalised ESS basis, showing the robust results that can be obtained from QIMHMC.

## 6 Results and discussion

**Fig 2:**
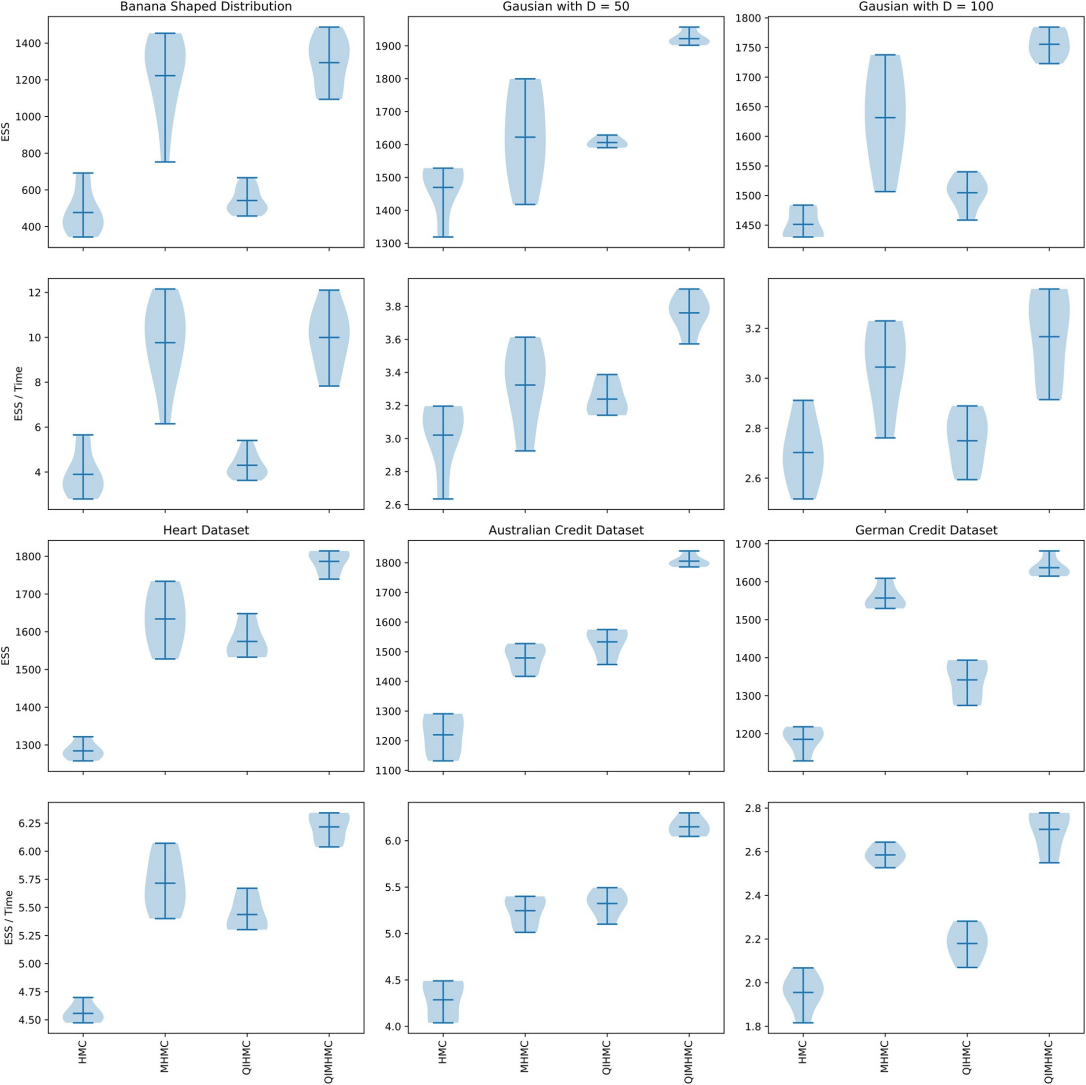
Results for the datasets over 10 runs of each method. For each dataset, the plots on the first row show the multivariate effective sample size and the plots on the second row show the multivariate effective sample size normalised by execution time (in seconds). For all the plots, the larger the value the better the method. The dark horizontal line in each violin plot represents the mean value over 10 runs of each algorithm.

We present the performance of the MCMC methods outlined above across all the targets distributions using different performance metrics in Fig 2 and Tables 2 to 4. In Fig 2, the plots on the first row for each dataset show the effective sample size, and the plots on the second row show the effective sample size normalised by execution time. The results are for the 10 runs of each algorithm. In Tables 2 to 4, each column corresponds with the results for a particular MCMC method. Values that are in **bold** indicate that the MCMC method outperforms on that particular metric.

**Table 2:** Banana shaped distribution results averaged over 10 runs. The time *t* is in seconds. The values in bold indicate that the particular method outperforms the other methods on that specific metric.

Banana shaped distribution				
Metric	HMC	MHMC	QIHMC	QIMHMC
Acceptance	77.42	**99.81**	75.52	99.10
ESS	476	1 222	542	**1 293**
ESS/*t*	3.89	9.76	4.30	**9.99**

**Table 3:** Multivariate Gaussian distribution results averaged over 10 runs. The time *t* is in seconds. The values in bold indicate that the particular method outperforms the other methods on that specific metric.

Gaussian with D = 50				
Metric	HMC	MHMC	QIHMC	QIMHMC
Acceptance	78.90	83.03	79.84	**89.3**
ESS	1 469	1 622	1 606	**1 921**
ESS/*t*	3.02	3.32	3.23	**3.76**
Gaussian with D = 100				
Acceptance	71.82	77.50	71.08	**80.24**
ESS	1 451	1 631	1 504	**1 755**
ESS/*t*	2.70	3.04	2.75	**3.17**

**Table 4:** Bayesian logistic regression results averaged over 10 runs. The time *t* is in seconds. The values in bold indicate that the particular method outperforms the other methods on that specific metric.

Heart dataset				
Metric	HMC	MHMC	QIHMC	QIMHMC
Acceptance	92.90	**99.00**	92.48	98.92
ESS	1 284	1 633	1 574	**1 786**
ESS/*t*	4.55	5.71	5.43	**6.21**
Australian credit dataset				
Acceptance	89.25	**96.70**	89.39	96.58
ESS	1 220	1 479	1 533	**1 805**
ESS/*t*	4.28	5.24	5.32	**6.15**
German credit dataset				
Acceptance	77.92	**89.08**	75.89	86.27
ESS	1 185	1 557	1 341	**1 636**
ESS/*t*	1.95	2.58	2.17	**2.70**

The execution time *t* in Fig 2 and Tables 2 to 4 is in seconds. The results in Tables 2 to 4 are the mean results over the 10 runs for each algorithm. We use the mean values over the 10 runs in Tables 2 to 4 to form our conclusions about the performance of the algorithms.

The results in Fig 2 and Tables 2 to 4 show that the proposed QIMHMC method outperforms all the other methods across the ESS and normalised ESS performance metrics and on all the targets. The outperformance improving with increasing dimensionality of the problem.

As expected, MHMC always outperforms HMC on all the targets and across all the metrics. This is in line with what has been previously observed [4, 6, 27]. MHMC and QIMHMC produce similar performance on the acceptance rate metric.

QIHMC outperforms HMC across all the targets. This confirms the results observed by Liu and Zhang [7] using different target posteriors to those considered in this paper. This shows the significant benefit that utilising a random mass can provide to the sampling properties of HMC based samplers. However, the real performance gains are only realised when the vol-of-vol parameter *α* has been appropriately tuned. The approach for tuning *α* is still an open research problem, which we aim to address in future work.

## 7 Conclusion

We present the Quantum-Inspired Magnetic Hamiltonian Monte Carlo (QIMHMC) method which employs a random mass matrix in the non-canonical dynamics of Magnetic Hamiltonian Monte Carlo (MHMC). This results in significant sampling improvements over MHMC. The new method is compared to Hamiltonian Monte Carlo (HMC), MHMC and Hamiltonian Monte Carlo with a random mass matrix. The methods are compared on the Banana shaped distribution, multivariate Gaussian distributions and on real world datasets modelled using Bayesian logistic regression.

The empirical results show that the new method outperforms all the other methods on a normalised effective sample size basis across all the targets. Furthermore, HMC with a random mass matrix outperforms vanilla HMC. This shows the significant benefit provided by using a random mass matrix to the sampling properties of HMC based samplers. A limitation of the method is the need to tune the vol-of-vol parameter. Although typically smaller values of the parameter improve the effective sample sizes, a more robust approach to the selection of the parameter is still required.

This work can be improved upon by establishing a heuristic or an automated approach to tune the vol-of-vol parameter. In addition, the tuning of the magnetic component could also be of interest. In future work, we plan to apply the proposed method to the inference of Bayesian neural networks using larger datasets such as MNIST.
